# Ultrasound localization of central vein catheter tip by contrast-enhanced transthoracic ultrasonography: a comparison study with trans-esophageal echocardiography

**DOI:** 10.1186/s13054-022-03985-3

**Published:** 2022-04-21

**Authors:** Francesco Corradi, Fabio Guarracino, Gregorio Santori, Claudia Brusasco, Guido Tavazzi, Gabriele Via, Silvia Mongodi, Francesco Mojoli, Raffaello Umberto Dario Biagini, Alessandro Isirdi, Federico Dazzi, Chiara Robba, Luigi Vetrugno, Francesco Forfori, UCARE research group, Maria Lidia Bologna, Alessandro Cardu, Laura Crocetti, Francesco Cundari, Elisa Del Frate, Samuele Ferrari, Alberto Laffi, Elena Marrucci, Marco Monfroni, Chiara Piagnani, Erika Taddei, Ludovica Tecchi, Sara Tempini, Debora Tognarelli, Carmelo Vullo

**Affiliations:** 1 Department of Surgical, Medical, Molecular Pathology and Critical Care Medicine, University of Pisa, Pisa, Italy; 2 Department of Anesthesia and Critical Care Medicine, Azienda Ospedaliero Universitaria Pisana, Pisa, Italy; 3 Department of Surgical Sciences and Integrated Diagnostics (DISC), University of Genoa, Genoa, Italy; 4 Anesthesia and Intensive Care Unit, E.O. Ospedali Galliera, Genoa, Italy; 5 Department of Clinical Surgical, Diagnostic and Pediatric Sciences, University of Pavia, Pavia, Italy; 6 Anaesthesia, Intensive Care and Pain Therapy, Fondazione IRCCS Policlinico San Matteo, Pavia, Italy; 7 Cardiac Anesthesia and Intensive Care, Fondazione Cardiocentro Ticino, Lugano, Switzerland; 8 Anesthesia and Intensive Care, Policlinico San Martino, IRCCS for Oncology and Neuroscience, Genoa, Italy; 9 Department of Medical, Oral and Biotechnological Sciences, University of Chieti-Pescara, Chieti, Italy; 10 Azienda Ospedaliero Universitaria Pisana, Via Paradisa, 2, 56124 Pisa, PI Italy

**Keywords:** Bubble test, Central venous catheterization, CVC misplacements, Cardiac surgery patients, Internal jugular vein cannulation, Chest radiography

## Abstract

**Background:**

To assess the usefulness of pre-operative contrast-enhanced transthoracic echocardiography (CE-TTE) and post-operative chest-x-ray (CXR) for evaluating central venous catheter (CVC) tip placements, with trans-esophageal echocardiography (TEE) as gold standard.

**Methods:**

A prospective single-center, observational study was performed in 111 patients requiring CVC positioning into the internal jugular vein for elective cardiac surgery. At the end of CVC insertion by landmark technique, a contrast-enhanced TTE was performed by both the apical four-chambers and epigastric bicaval acoustic view to assess catheter tip position; then, a TEE was performed and considered as a reference technique. A postoperative CXR was obtained for all patients.

**Results:**

As per TEE, 74 (67%) catheter tips were correctly placed and 37 (33%) misplaced. Considering intravascular and intracardiac misplacements together, they were detected in 8 patients by CE-TTE via apical four-chamber view, 36 patients by CE-TTE via epigastric bicaval acoustic view, and 12 patients by CXR. For the detection of catheter tip misplacement, CE-TTE via epigastric bicaval acoustic view was the most accurate method providing 97% sensitivity, 90% specificity, and 92% diagnostic accuracy if compared with either CE-TTE via apical four-chamber view or CXR. Concordance with TEE was 79% (p < 0.001) for CE-TTE via epigastric bicaval acoustic view.

**Conclusions:**

The concordance between CE-TTE via epigastric bicaval acoustic view and TEE suggests the use of the former as a standard technique to ensure the correct positioning of catheter tip after central venous cannulation to optimize the use of hospital resources and minimize radiation exposure.

**Supplementary Information:**

The online version contains supplementary material available at 10.1186/s13054-022-03985-3.

## Background

Central venous catheter (CVC) placement is a common procedure in the operating room and intensive care unit (ICU), for CVC allows the administration of hypertonic and vesicant drugs with better control of infusion velocity [1]. In upper extremity cannulation, the CVC tip should be placed in the last 3 cm of the superior vena cava (SVC) before its junction to right atrium (SVC-RA) [2]. The evaluation of catheter tip position before CVC is recommended as a misplacement may result in severe complications whether being within cardiac chambers (arrhythmias/cardiac wall damage) [2] or into the upper-veins system (vein endothelium damage with extravasation, and pleural effusion, or thrombosis) [3, 4]. CVC-related-thrombosis is reported between 30 and 70% of cases [3–5] and represents a frequent, although largely under-recognized, complication potentially causing life-threatening sequelae such as pulmonary embolism and catheter-related sepsis [3, 4, 6–8].

A post-procedural chest-x-ray (CXR) is routinely obtained after CVC cannulation of the upper extremity according to current guidelines [9]. However, CXR is limited by the indirect visualization of vessels and the CVC tip positioning is inferred from its projection on anatomical structures, such as the carina or dorsal vertebrae [10]. Despite its wide use, CXR presents low accuracy when compared to transoesophageal echocardiography (TEE), assumed as the gold standard [10]. TEE represents the only bedside tool that can directly visualize CVC tip at the SVC-RA junction [11], but it is invasive, time-consuming, and requires specific competences. Transthoracic echocardiography (TTE), with contrast-enhancement (CE), has been proposed as an alternative to CXR in detecting CVC positioning with high accuracy [12]. Nevertheless, TTE-CE is not widely used also considering some previous conflicting results [13] and only comparison between TTE and CXR has been reported [14].

The aim of the present study was to assess the diagnostic accuracy of CEUS-TTE, by two different ultrasound acoustic views (apical four-chambers and subcostal), and CXR for the detection of CVC tip misplacements in comparison with TEE as gold standard.

## Materials and methods

### Study population

This study was conducted from April 1 to October 31, 2019, in the cardiac surgery operating room of the Pisa University Hospital. The study protocol was approved by the Institutional Ethics Committee (CEAVNO approval number 25585/2019). Inclusion criteria were adult age and elective cardiac surgery with undergoing TEE as per clinical indications. Exclusion criteria were patient’s inability to sign an informed consent or no CVC in site.

### Methods

The CVCs were placed by different physicians following orotracheal intubation and before surgery. Non-tunnelled, 7F dual-lumen, 20-cm length CVCs (BD Careflow; Becton Dickinson Critical Care Systems, Franklin Lakes, NJ) were inserted percutaneously into the right internal jugular vein, by standard Seldinger technique using anatomical landmarks, without the use of either fluoroscopy or intraoperative ultrasound guidance. After CVC insertion, a skilled cardio-anaesthesiologist, not directly involved in the CVC placement, performed all echocardiographic examinations to assess the correct CVC position.

### Echocardiographic techniques

**Fig. 1:**
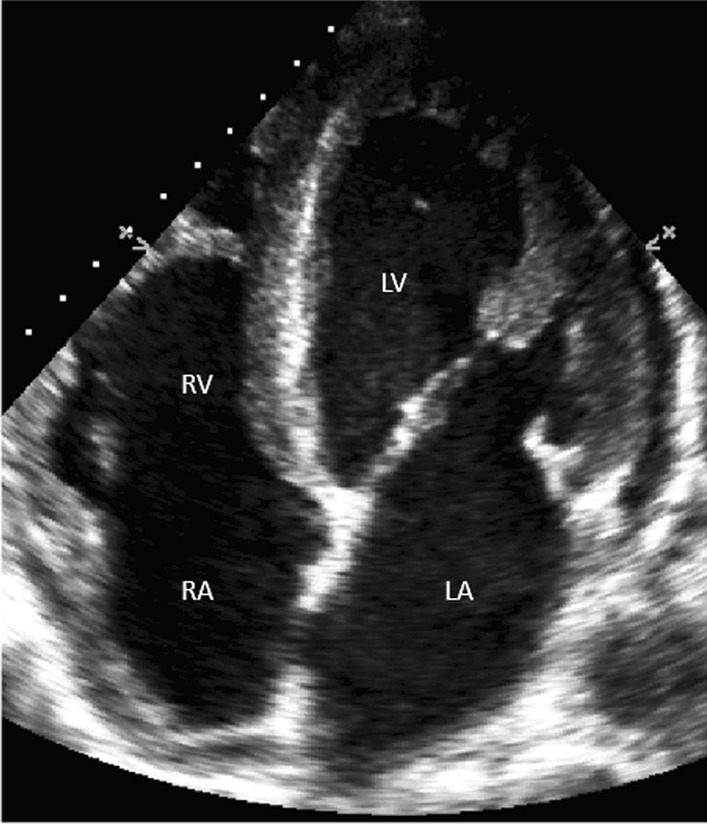
Heart ultrasounography. Apical four chamber view. *RA* right atrium, *RV* right ventricle, *LA* left atrium, *LV* left ventricle

**Fig. 2:**
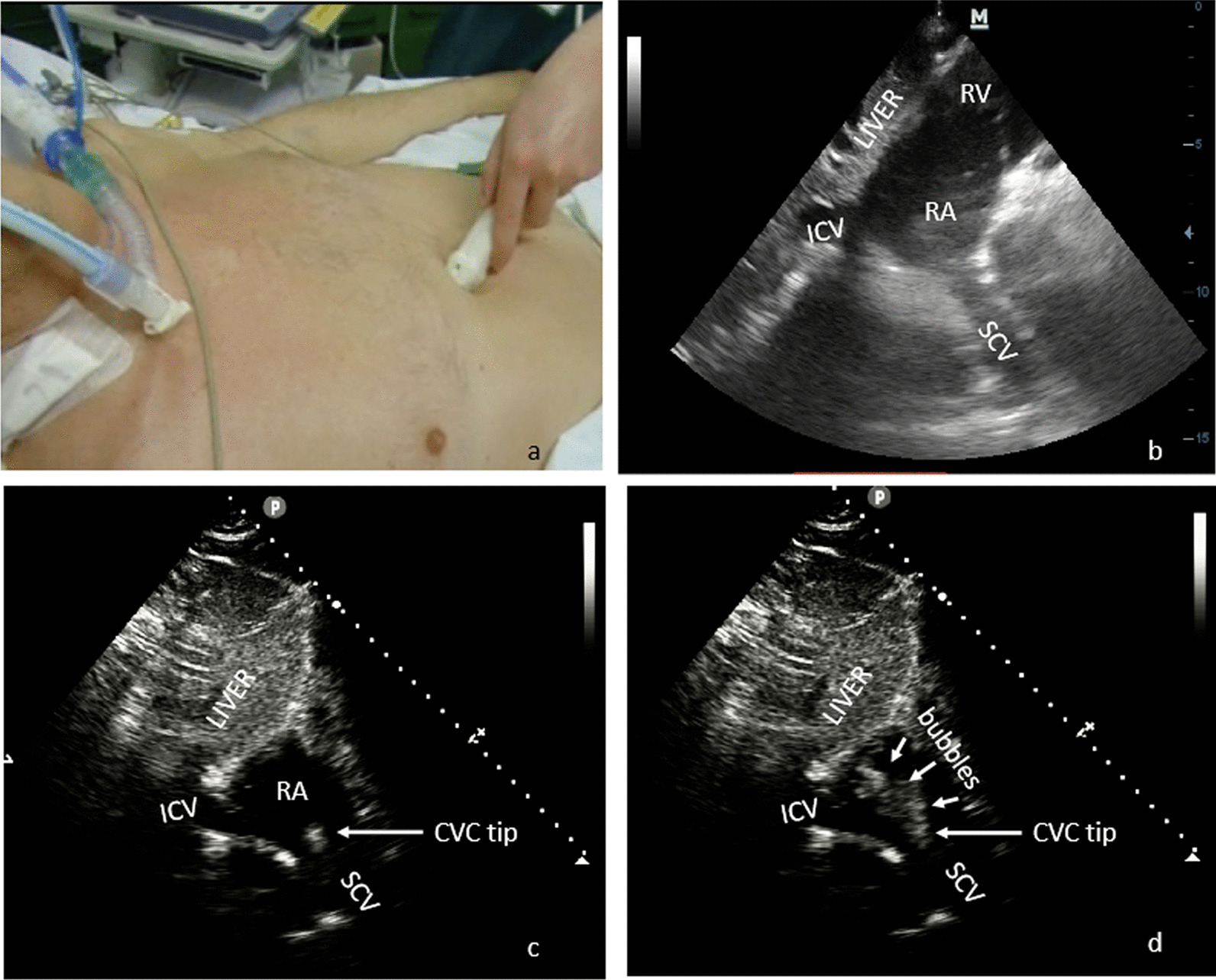
**a** Probe orientation in the epigastric bicaval acoustic window. **b** B-mode short-axis bicaval view, **c** CVC tip, **d** microbubbles solution injected as a bolus through the catheter directly showing the catheter tip and CE exit point. *ICV* inferior cava vein, *SVC* superior vena cava, *RA* right atrium, *RV* right ventricle, *CVC tip* central venous catheter tip

Examinations were performed by using an iE33 ultrasound system (Philips Ultrasound, Bothell, WA, USA) with a 2.5–3.5 MHz sector probe. The TTE apical four-chambers (Fig. 1) and short-axis subcostal bicaval views were scanned (Fig. 2a, b). A standard bubble-study with rapid flush of agitated saline was used [15] to confirm the correct identification of the CVC tip (Fig. 2c, d). The contrast medium was a saline-air mixture with two 10-mL syringes containing 8 mL of saline, 1 mL of air and 1 mL of blood each. A homogeneous solution was obtained by mixing with a three-way stopcock and a 3-mL bolus was injected through the catheter. Microbubbles were identified to verify the catheter tip position in three steps: (i) during apical four-chamber view; (ii) during subcostal view scanning; (iii) during TEE examination.

With the apical four-chambers view, the CVC was considered correctly positioned when a laminar saline swirl entering the right atrium was observed immediately after saline injection as previously described [16], because this method does not allow direct visualization of the SVC-RA junction. Misplacement was defined as catheter tip too distal in RA (Additional file 1). With the subcostal view, the correct catheter position was inferred from direct detection of catheter tip confirmed by the visualization of the CE exit point at the SVC-RA junction (Additional file 2). Misplacements were defined as catheter tip too distal in RA or not directly visualized by B-mode ultrasound, despite the appearance of a laminar jet flow from the SVC.

**Fig. 3:**
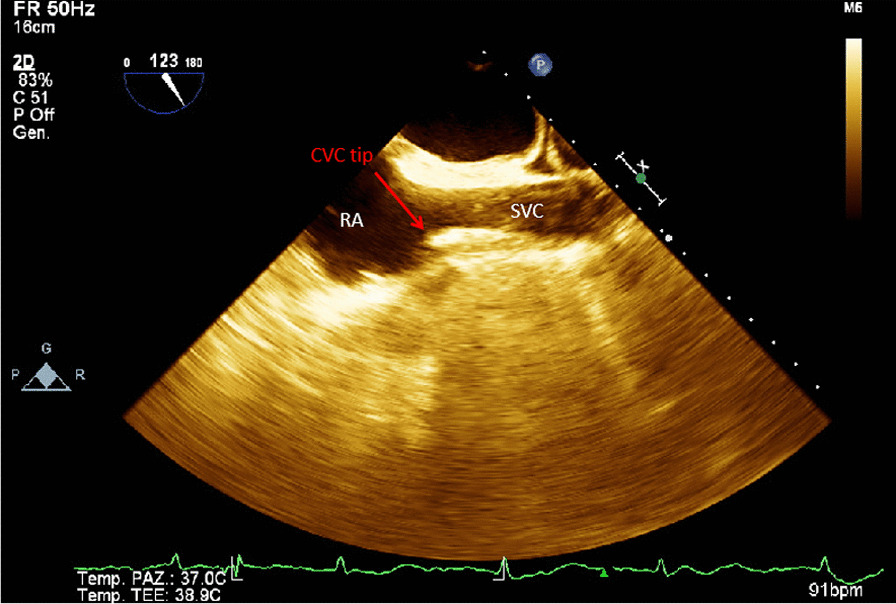
TEE probe inserted at a mid-oesophageal position, turned clockwise and rotated to 123° to produce a mid-oesophageal SVC-RA junction visualization. Red arrow: central venous catheter tip at SCV-RA junction. *SVC* superior vena cava, *RA* right atrium, *CVC tip* central venous catheter tip

By TEE probe via mid-oesophageal bicaval view (90°–10°) (Fig. 3), the CVC was considered correctly positioned when the CVC tip was identified in the last 3 cm of the SVC measured from cresta terminalis (CT). Misplacement was defined as the catheter tip too distal in RA or too proximal in the SVC (more than 3 cm above the cresta terminalis) or not directly visualized by B-mode ultrasound, despite the appearance of a laminar jet flow coming from the SVC. The catheter was identified as two closely spaced, parallel, bright hyperechoic lines surrounding the dark fluid-filled lumen. (Additional file 3).

Post-operative CXR results were also collected in all patients as required by the on-duty physician for clinical purposes. By CXR the CVC was considered correctly positioned when the CVC tip was identified in the SVC/RA junction defined as the apex of the concave shadow formed by the superimposition of the distal SVC on the right atrium. A senior radiologist examined all the plain film radiographs.

### Statistical analysis

A sample size of at least 94 patients was required by assuming sensitivity = 0.90, delta = 0.10, alpha = 0.05 and power = 0.80. A true positive result was defined as the judgment of correct placement that was eventually confirmed by TEE. A true negative result was defined as the judgment of an incorrect placement that was confirmed by TEE. False positive and negative results were defined accordingly. Sensitivity, specificity, positive/negative predictive values and likelihood ratios, diagnostic accuracy/concordance between different techniques and TEE were calculated. For the latter, Cohen’s *k* statistics were used, with 0–0.20 indicated slight agreement, 0.21–0.40 fair agreement, 0.41–0.60 moderate agreement, 0.61–0.80 substantial agreement, and 0.81–1.00 almost perfect agreement [17]. ROC curves were used to evaluate the diagnostic accuracy of echocardiographic techniques and chest-x-rays assuming transesophageal echocardiography as the gold standard. For each ROC curve, the area under the curve (AUC) and the corresponding 95% confidence interval (CI) was calculated. The comparisons between the AUC of two ROC curves were performed by applying the De Long test and bootstrap at 2000 replicates. Continuous variables were expressed as median and inter-quartile range (IQR) or mean ± standard deviation as appropriate. Statistical significance was set at *p* < 0.05 with two-sided tests. All data were analyzed with SPSS (version 20, IBM Corporation, New York, NY) and R statistical environment (version 4.0.3, R Foundation for Statistical Computing, Vienna, Austria).

## Results

We initially considered 112 patients, but in one case TEE was not able to show CVC tip position due to massive enlargement of the ascending aorta; thus, we finally analyzed the data of 111 patients: 69 males (62%) with median age of 70 (IQR: 60–75) years.

TEE exams were obtained in all cases whereas CE-TTE was not obtainable via apical four-chamber view in 5 patients (5%) and via subcostal bicaval view in 3 (2.7%), due to difficult visualization of the acoustic window. The median body mass index (BMI) was 25.5 (IQR: 23–28) kg/m^2^ and was higher in those patients in whom CE-TTE was not feasible (29 vs. 25 kg/m^2^; *p* = 0.017).

In our population, 74 (67%) TEE catheter tips were correctly placed at the SVC-RA junction within 3 cm from cresta terminalis, while 37 (33%) were misplaced. Concerning these misplaced catheter tips, 18 (49%) were too proximal in the SVC, 17 (46%) were too distal in the right atrium, and in 2 cases (5%) catheters were misplaced in vessels other than SCV (pulmonary artery in one case and homolateral subclavian vein in the other). None of the patients had the CVC tip positioned in the inferior vena cava.

Apical four-chamber CE-TTE misdiagnosed 32 cases; of these, 4 were wrongly identified as misplaced and 28 wrongly identified as correctly placed. However, apical four-chamber CE-TTE detected 8 out of 37 misplacements identified by TEE, all distally located.

Of the 5 non-feasible CE-TTE cases, one had the CVC in the RA whereas 4 were correctly positioned at the SVC-RA junction.

Subcostal bicaval CE-TTE diagnosed 36 out of 37 misplacements, including 16 in the RA, 19 in the SCV, and 1 in the pulmonary artery. The catheter distally located in the pulmonary artery was classified as misplaced because neither tip nor contrast exit point were directly visualized. Seven catheters lying at a median distance of 2.5 cm (IQR: 1.5–3 cm) from the CT were classified as misplaced even if they were not. By this technique, were correctly visualized 64 catheters tips located in the SVC-RA junction at a median distance of 1 cm (IQR: 0–1.5 cm) from the CT. Seven cases were erroneously visualized as wrong positioned. The 3 non-feasible cases due to poor acoustic window, were all correctly located in the SCV.

Post-operative CXR detected 12 out of 37 misplacements including 4 in the RA, 6 in the proximal SCV, and 2 in the homolateral subclavian vein or pulmonary artery, respectively. Five cases were erroneously interpreted as wrong positioned even if they were not. Twenty-five CXR did not detect 13 catheter tips in the RA and 12 located in the proximal SCV. CXR was feasible in all patients.

The diagnostic accuracy of the different techniques compared with the reference TEE are reported in Additional file 4. The concordance between TEE and CE-TTE subcostal view was strong (*k* = 0.79), whereas the concordance was poor when compared with the CE-TTE 4-chambers view or CXR (*k* = 0.17 and *k* = 0.27, respectively).

**Fig. 4:**
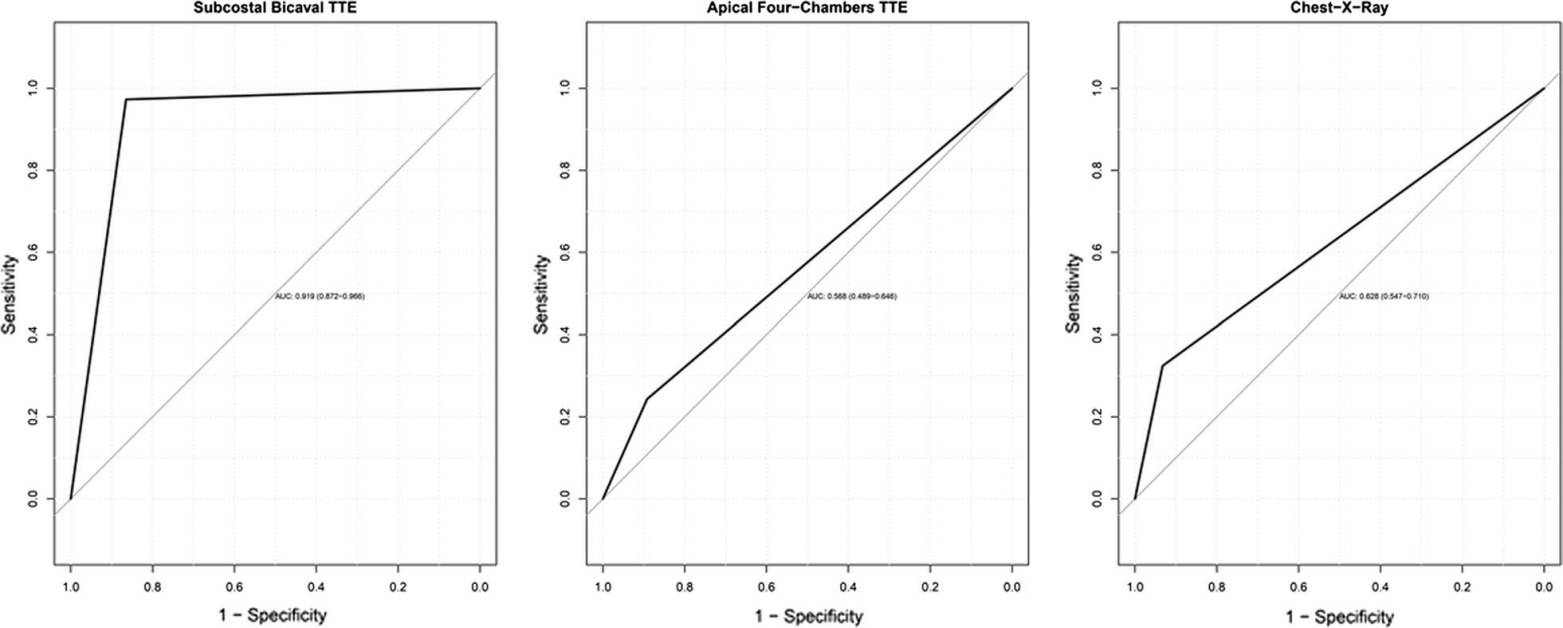
ROC curves for subcostal bicaval transthoracic echocardiography (subcostal Bicaval TTE), apical four-chamber transthoracic echocardiography (apical four-chamber TTE) and chest-x-ray (CXR), with transesophageal echocardiography assumed as reference. *AUC* area under curve (95% confidence interval)

The ROC curves and the corresponding AUC for CE-TTE subcostal view, apical four-chambers view and CXR are shown in Fig. 4. By comparing ROC curves, significant differences were found for subcostal bicaval TTE versus apical four-chamber TTE (*p* < 0.001) and subcostal bicaval TTE versus CXR (*p* < 0.001), whereas no statistical significance was observed for apical four-chamber TTE versus CXR (*p* = 0.388).

## Discussion

The main results of the study are that (1) the incidence of catheter misplacements was 33%, with half of them due to a tip position proximal to SCV-RA junction; (2) CE-TTE subcostal bicaval view was the best transthoracic echocardiographic approach providing 92% diagnostic accuracy and 79% concordance with CE-TEE; (3) CXR, as well as CE-TTE four-chambers view, had a limited value in the detection of misplacements with a poor concordance with CE-TEE.

A CVC tip positioned at the transition from the SVC to the right atrium, lying parallel to the major axis of the vessel, is considered as the “ideal position” however difficult to obtain. CVC-related venous thrombosis has a high prevalence when the catheter tip is positioned in the superior upper third of the SVC or in the brachiocephalic vein [18] and represents a major cause of late complications finally resulting in SVC syndrome, infections and pulmonary embolism, occurring mostly in oncologic frail patients [19, 20].

In our study, the incidence of catheter misplacements was higher than previously reported [14, 21]. Possible explanations are the following: first, in most of other studies [21] chest radiography was choose as reference standard even if inaccurate for tip location [14]; second, in our center catheters were placed by standard Seldinger technique using anatomical landmarks, without use of intraoperative ultrasound guidance or navigation procedures; third, 20 cm of catheter length were inserted in the right internal jugular vein, because this is the current clinical practice in our center. Altogether, the above methodological issues might have increased the rate of misplacements, but it seems unlikely that they might have affected the results.

TEE is considered the gold standard to assess the correct position of CVC though it is not free from complications related to probe insertion and should not be therefore used for this purpose. The strong concordance between TEE and TTE subcostal bicaval view (*k* = 0.79) is clinically important. Indeed, TTE subcostal bicaval view is non-invasive, frequently obtainable, and based on direct visualization of the CVC tip at the SVC-RA junction. In seven of our cases, we classified the catheters as misplaced even if their tips were correctly placed at a median distance of 2.5 cm (IQR: 1.5–3 cm) from the CT. This was presumably due to the difficulty of visualizing the CVC tip when lying in the proximal portions of the SVC. However, our results show that the probability of classifying a correctly placed catheter as misplaced is 9.7 time lower than the probability of classifying a misplaced catheter as correctly placed.

The CE-TTE four-chambers view allowed showing the correct position of catheter tip in the SVC through the “bubble-test” [22]; additionally, a low concordance with CE-TEE (*k* = 0.17) was observed. CE-TTE four-chambers view misdiagnosed catheter position in 32 patients. These findings may have a twofold explanation. First, bubbles reach the RA even in cases of intravascular misplacements. Second, it may be difficult to differentiate between bubbles originating within the RA from bubbles reaching the RA, because CE-TTE four-chambers view does not allow direct visualization of the SVC-RA junction. Indeed, by this technique, intra-atrial misplacements were more readily detected compared to extra-atrial misplacements and the high number of extra-atrial misplacements depicted by TEE led to a lower sensitivity than previously described [16].

Evaluation of different patterns of jet flow (laminar or turbulent) and bubbles transition-times have been suggested [12] to define if the arrival of the bubbles was associated with correct positioning. We decided not to adopt this method, because in our experience transition-times may be dependent on (a) patient hemodynamic, (b) length and diameter of the catheter, (c) CVC insertion point, (d) infusion velocity of contrast media, and (e) assessment difficulties (no commercially available device to synchronize “push-to-bubble time”). A previous study [22] investigating the “qualitative” bubble test, defined as the complete RA opacization versus “few or no bubbles” detection, reported that the complete opacization of RA occurred in half of the patients with CVC misplacements, claiming its low diagnostic accuracy. Furthermore, another study has questioned the usefulness of the proposed cut-off transition times to confirm central catheters tips position [23].

Some ultrasound-based tip navigation methods suggested direct visualization of the guidewire during the insertion procedure [24, 25]. These methods allow advancing the guidewire with catheter insertion, and misplacements can be quickly recognized. However, this technique can be applied only during real-time ultrasound-guided cannulations, whereas many physicians still use landmark procedures [26]. On the contrary, tip location methods allow checking the catheter tip when already in place. Thus, tip navigation does not replace tip location, which should be assumed as complementary methods [14].

CXR also showed low concordance with CE-TEE (*k* = 0.29), as it led to a catheter position misdiagnosis in 27% of cases. These results can be explained considering that the SCV-RA junction cannot be directly visualized by bedside CXR and catheter tip positioning is presumed on the basis of its projection on other fixed anatomical structures taken as radiological landmarks, none of them being 100% reliable [27, 28]. Likewise, the detection of SVC/RA junction by the shadow of SVC on the RA can be difficult [29].

In recent years, the potential role of transthoracic ultrasound has been investigated as an alternative to CXR in the detection of catheter positioning [12, 13, 15, 16, 30–32], but not all studies confirmed these results[13], mainly because of methodological differences (detection of tip positioning vs. bubble test or subcostal bicaval vs. apical four-chambers scanning view) and lack of a reliable comparator (TEE). This is the reason why, although the ultrasound-guided CVC insertion has been widely accepted to improve success and safety of the procedures [26, 33, 34], the CVC position confirmation by CE-TTE has not yet obtained widespread dissemination [35] and in many centers CXR is still considered the reference confirmatory test [31].

To the best of our knowledge, this is the first study rigorously comparing CXR, TTE with TEE. Our results confirm that CE-TTE via short-axis subcostal bicaval view, but neither CE-TTE via apical four-chambers view nor CXR, represents a reliable tool to identify CVC tip misplacements when compared with CE-TEE. CE-TTE via apical four-chambers view is usually used only to rule-out RA misplacements, but in our population the sensitivity was only 50% and it appears to be unreliable in ruling CVC lying proximally in the SCV. Post-operative bedside CXR is always feasible, but its usefulness is limited by its poor diagnostic accuracy.

Limitations: First, our study was conducted in elective anesthetized and pharmacologically paralyzed patients undergoing cardiac surgery. This controlled setting may have influenced the results of the study making TTE scans easier to acquire especially in the subcostal views. Thus, our results may not be directly applied in other clinical contexts such as awake, non-paralyzed, obese patients, emergency, or trauma. Second, all CVCs were inserted percutaneously without ultrasound guide assistance, thus a perioperative vascular scan of latero-cervical, supraclavicular and infraclavicular fossae to identify errant catheters [26] was not performed. However, we believe that this limitation did not affect the results of the study because vascular ultrasound, however useful for aiding insertion and detecting misplacements in neck veins [26], is of limited value in confirming tip placement because not suitable for the imaging of SCV. Third, we did not use right atrial electrocardiography during CVC positioning because by this technique it is only possible to detect the tip within the RA but not its exact location within the vascular system. Fourth, only one skilled cardio-anesthesiologist not involved in the CVC placement performed all the examinations to avoid inter-rater variability. Therefore, this source of variability was not considered, and the study was focused on the variability between techniques only. Finally, although the number of patients exceeded the sample size estimation, further single- or multi-center studies including large numbers of patients are needed to confirm our results.

## Conclusions

The present study suggests that CE-TTE via the subcostal bicaval short-axis view may represent an accurate technique in detecting CVC misplacements after internal jugular vein cannulation, with an accuracy similar to that of the TEE gold standard. The use of CXR should be restricted to those cases where ultrasound examinations are not feasible due to technical limitations.

## Supplementary Information

**Additional file 1.** Transthoracic apical four-chambers acoustic window showing a clear jet flow coming from the right atrium immediately after agitated saline injection, corresponding to aberrant central line tip positioning.

**Additional file 2.** Transthoracic subcostal bicaval acoustic window showing superior vena cava– right atrium junction: a laminar flow appears from the superior vena cava immediately after agitated saline injection, corresponding to correct central line tip positioning.

**Additional file 3.** TEE bicaval view by which catheter tip was identified as two closely spaced, parallel, bright hyperechoic lines surrounding the dark fluid-filled lumen and CE exit point at SVC-RA junction.

**Additional file 4.** Comparative results of contrast-enhanced transthoracic echocardiography and chest-X-ray with transesophageal echocardiography assumed as reference. Subcostal Bicaval Transthoracic Echocardiography (TTE); Apical Four-Chamber Transthoracic Echocardiography; CXR: Chest-X-Ray; PPV: Positive Predictive Value; NPV: Negative Predictive Value; LR: Likelihood Ratio.

## Data Availability

Data will be made available by the authors for global collaboration on reasonable request, within the national restrictions imposed by privacy laws and ethics.

## Abbreviations

CE: Contrast-enhanced ultrasound
TTE: Transthoracic echocardiography
CXR: Chest-x-ray
CVC: Central venous catheter
TEE: Transesophageal echocardiography
SVC: Superior vena cava
RA: Right atrium
SVC-RA: Superior vena cava to right atrium junction

## References

[1] Kolikof J, Peterson K, Baker AM. Central Venous Catheter. StatPearls [Internet]. Treasure Island (FL): StatPearls Publishing; 2021 [cited 2021 Sep 14]. http://www.ncbi.nlm.nih.gov/books/NBK557798/32491730

[2] Fletcher SJ, Bodenham AR (2000). Safe placement of central venous catheters: where should the tip of the catheter lie?. Br J Anaesth.

[3] Timsit JF, Farkas JC, Boyer JM, Martin JB, Misset B, Renaud B (1998). Central vein catheter-related thrombosis in intensive care patients: incidence, risks factors, and relationship with catheter-related sepsis. Chest.

[4] Raad II, Luna M, Khalil SA, Costerton JW, Lam C, Bodey GP (1994). The relationship between the thrombotic and infectious complications of central venous catheters. JAMA.

[5] Hoch JR (1997). Management of the complications of long-term venous access. Semin Vasc Surg.

[6] ECG and echocardiographic diagnosis of pulmonary thromboembolism associated with central venous lines - PubMed [Internet]. [cited 2021 Sep 14]. https://pubmed.ncbi.nlm.nih.gov/7574859/

[7] Dollery CM, Sullivan ID, Bauraind O, Bull C, Milla PJ (1994). Thrombosis and embolism in long-term central venous access for parenteral nutrition. Lancet.

[8] Heiselman D (1994). Nosocomial bloodstream infections in the critically ill. JAMA.

[9] Frykholm P, Pikwer A, Hammarskjöld F, Larsson AT, Lindgren S, Lindwall R (2014). Clinical guidelines on central venous catheterisation. Swedish Society of Anaesthesiology and Intensive Care Medicine. Acta Anaesthesiol Scand.

[10] Wirsing M, Schummer C, Neumann R, Steenbeck J, Schmidt P, Schummer W (2008). Is traditional reading of the bedside chest radiograph appropriate to detect intraatrial central venous catheter position?. Chest.

[11] Andropoulos DB, Stayer SA, Bent ST, Campos CJ, Bezold LI, Alvarez M (1999). A controlled study of transesophageal echocardiography to guide central venous catheter placement in congenital heart surgery patients. Anesth Analg.

[12] Vezzani A, Brusasco C, Palermo S, Launo C, Mergoni M, Corradi F (2010). Ultrasound localization of central vein catheter and detection of postprocedural pneumothorax: an alternative to chest radiography. Crit Care Med.

[13] Weekes AJ, Keller SM, Efune B, Ghali S, Runyon M (2016). Prospective comparison of ultrasound and CXR for confirmation of central vascular catheter placement. Emerg Med J.

[14] Lamperti M, Biasucci DG, Disma N, Pittiruti M, Breschan C, Vailati D (2020). European Society of Anaesthesiology guidelines on peri-operative use of ultrasound-guided for vascular access (PERSEUS vascular access). Eur J Anaesthesiol.

[15] Gekle R, Dubensky L, Haddad S, Bramante R, Cirilli A, Catlin T (2015). Saline flush test: can bedside sonography replace conventional radiography for confirmation of above-the-diaphragm central venous catheter placement?. J Ultrasound Med.

[16] Cortellaro F, Mellace L, Paglia S, Costantino G, Sher S, Coen D (2014). Contrast enhanced ultrasound vs chest x-ray to determine correct central venous catheter position. Am J Emerg Med.

[17] Landis JR, Koch GG (1977). The measurement of observer agreement for categorical data. Biometrics.

[18] Caers J, Fontaine C, Vinh-Hung V, De Mey J, Ponnet G, Oost C (2005). Catheter tip position as a risk factor for thrombosis associated with the use of subcutaneous infusion ports. Support Care Cancer.

[19] Ray S, Stacey R, Imrie M, Filshie J (1996). A review of 560 Hickman catheter insertions. Anaesthesia.

[20] Eastridge BJ, Lefor AT (1995). Complications of indwelling venous access devices in cancer patients. J Clin Oncol.

[21] Smit JM, Raadsen R, Blans MJ, Petjak M, Van de Ven PM, Tuinman PR (2018). Bedside ultrasound to detect central venous catheter misplacement and associated iatrogenic complications: a systematic review and meta-analysis. Crit Care.

[22] Meggiolaro M, Scatto A, Zorzi A, Roman-Pognuz E, Lauro A, Passarella C (2015). Confirmation of correct central venous catheter position in the preoperative setting by echocardiographic “bubble-test”. Minerva Anestesiol.

[23] Gidaro A, Casella F, Lugli F, Cogliati C, Calloni M, Samartin F, et al. Contrast enhanced ultrasound as a new tool to estimate the performance of midline catheters in the single patient. J Vasc Access. 2021;11297298211034628. 10.1177/11297298211034629 34289731

[24] Bedel J, Vallée F, Mari A, Riu B, Planquette B, Geeraerts T (2013). Guidewire localization by transthoracic echocardiography during central venous catheter insertion: a periprocedural method to evaluate catheter placement. Intensive Care Med.

[25] Kim S-C, Heinze I, Schmiedel A, Baumgarten G, Knuefermann P, Hoeft A (2015). Ultrasound confirmation of central venous catheter position via a right supraclavicular fossa view using a microconvex probe: an observational pilot study. Eur J Anaesthesiol.

[26] Schmidt GA, Blaivas M, Conrad SA, Corradi F, Koenig S, Lamperti M (2019). Ultrasound-guided vascular access in critical illness. Intensive Care Med.

[27] Aslamy Z, Dewald CL, Heffner JE (1998). MRI of central venous anatomy: implications for central venous catheter insertion. Chest.

[28] Reynolds N, McCulloch AS, Pennington CR, MacFadyen RJ (2001). Assessment of distal tip position of long-term central venous feeding catheters using transesophageal echocardiology. JPEN J Parenter Enteral Nutr.

[29] Hsu J-H, Wang C-K, Chu K-S, Cheng K-I, Chuang H-Y, Jaw T-S (2006). Comparison of radiographic landmarks and the echocardiographic SVC/RA junction in the positioning of long-term central venous catheters. Acta Anaesthesiol Scand.

[30] Vezzani A, Manca T, Brusasco C, Santori G, Valentino M, Nicolini F (2014). Diagnostic value of chest ultrasound after cardiac surgery: a comparison with chest X-ray and auscultation. J Cardiothorac Vasc Anesth.

[31] Duran-Gehring PE, Guirgis FW, McKee KC, Goggans S, Tran H, Kalynych CJ (2015). The bubble study: ultrasound confirmation of central venous catheter placement. Am J Emerg Med.

[32] Maury E, Guglielminotti J, Alzieu M, Guidet B, Offenstadt G (2001). Ultrasonic examination: an alternative to chest radiography after central venous catheter insertion?. Am J Respir Crit Care Med.

[33] Brusasco C, Corradi F, Zattoni PL, Launo C, Leykin Y, Palermo S (2009). Ultrasound-guided central venous cannulation in bariatric patients. Obes Surg.

[34] Vezzani A, Manca T, Brusasco C, Santori G, Cantadori L, Ramelli A (2017). A randomized clinical trial of ultrasound-guided infra-clavicular cannulation of the subclavian vein in cardiac surgical patients: short-axis versus long-axis approach. Intensive Care Med.

[35] Ablordeppey EA, Drewry AM, Theodoro DL, Tian L, Fuller BM, Griffey RT (2019). Current practices in central venous catheter position confirmation by point of care ultrasound: a survey of early adopters. Shock.

