# Supplementary material for: Ultrasound localization of central vein catheter tip by contrast-enhanced transthoracic ultrasonography: a comparison study with trans-esophageal echocardiography
Source: Crit Care. 2022 Apr 21;26:113. doi: 10.1186/s13054-022-03985-3 (PMC9027702; doi:10.1186/s13054-022-03985-3)
Supplement: Supplementary file 4 — Additional file 4. Comparative results of contrast-enhanced transthoracic echocardiography and chest-X-ray with transesophageal echocardiography assumed as reference. Subcostal Bicaval Transthoracic Echocardiography (TTE); Apical Four-Chamber Transthoracic Echocardiography; CXR: Chest-X-Ray; PPV: Positive Predictive Value; NPV: Negative Predictive Value; LR: Likelihood Ratio. [file 13054_2022_3985_MOESM4_ESM.docx]

| **Technique** | **Sensitivity** | **Specificity** | **PPV** | **NPV** | **LR+** | **LR-** | **Accuracy** | **Cohen *k*** | **p** |
| --- | --- | --- | --- | --- | --- | --- | --- | --- | --- |
| Subcostal Bicaval TTE | 0.97 | 0.90 | 0.83 | 0.98 | 9.7 | 0.03 | 0.92 | 0.79 | <0.01 |
| Apical Four-Chamber TTE | 0.22 | 0.94 | 0.66 | 0.70 | 3.66 | 0.83 | 0.70 | 0.17 | 0.02 |
| Chest-X-Ray | 0.32 | 0.93 | 0.70 | 0.73 | 4.57 | 0.73 | 0.73 | 0.29 | 0.01 |
